# Identification of PPARgamma Partial Agonists of Natural Origin (I): Development of a Virtual Screening Procedure and In Vitro Validation

**DOI:** 10.1371/journal.pone.0050816

**Published:** 2012-11-30

**Authors:** Laura Guasch, Esther Sala, Anna Castell-Auví, Lidia Cedó, Klaus R. Liedl, Gerhard Wolber, Markus Muehlbacher, Miquel Mulero, Montserrat Pinent, Anna Ardévol, Cristina Valls, Gerard Pujadas, Santiago Garcia-Vallvé

**Affiliations:** 1 Grup de Recerca en Nutrigenòmica, Departament de Bioquímica i Biotecnologia, Universitat Rovira i Virgili, Tarragona, Catalonia, Spain; 2 Department of Theoretical Chemistry, Faculty of Chemistry and Pharmacy, Center for Molecular Biosciences, Leopold-Franzens-University Innsbruck, Innsbruck, Austria; 3 Institute of Pharmacy, Freie Universitaet Berlin, Berlin, Germany; 4 Department of Psychiatry and Psychotherapy, University of Erlangen, Erlangen, Germany; 5 Centre Tecnològic de Nutrició i Salut (CTNS), TECNIO, CEICS, Reus, Catalonia, Spain; Semmelweis University, Hungary

## Abstract

**Background:**

Although there are successful examples of the discovery of new PPARγ agonists, it has recently been of great interest to identify new PPARγ partial agonists that do not present the adverse side effects caused by PPARγ full agonists. Consequently, the goal of this work was to design, apply and validate a virtual screening workflow to identify novel PPARγ partial agonists among natural products.

**Methodology/Principal Findings:**

We have developed a virtual screening procedure based on structure-based pharmacophore construction, protein-ligand docking and electrostatic/shape similarity to discover novel scaffolds of PPARγ partial agonists. From an initial set of 89,165 natural products and natural product derivatives, 135 compounds were identified as potential PPARγ partial agonists with good ADME properties. Ten compounds that represent ten new chemical scaffolds for PPARγ partial agonists were selected for *in vitro* biological testing, but two of them were not assayed due to solubility problems. Five out of the remaining eight compounds were confirmed as PPARγ partial agonists: they bind to PPARγ, do not or only moderately stimulate the transactivation activity of PPARγ, do not induce adipogenesis of preadipocyte cells and stimulate the insulin-induced glucose uptake of adipocytes.

**Conclusions/Significance:**

We have demonstrated that our virtual screening protocol was successful in identifying novel scaffolds for PPARγ partial agonists.

## Introduction

**Figure 1:**
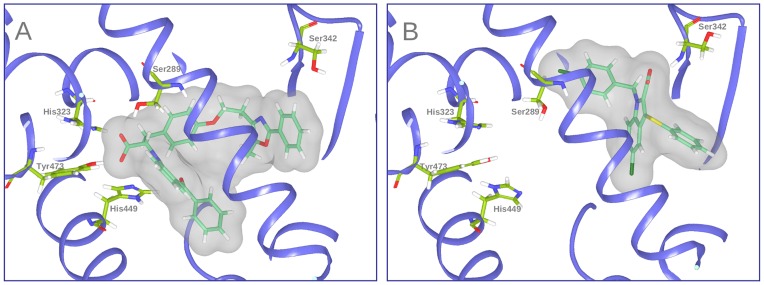
Binding models of (A) the PPARγ full agonist Farglitazar (crystal structure 1FM9) and (B) the PPARγ partial agonist nTZDpa (crystal structure 2Q5S). Important binding residues are depicted as wireframes with green carbon atoms. Oxygen, nitrogen, and hydrogen atoms are colored red, blue and white, respectively.

Peroxisome proliferator-activated receptors (PPARs) are members of the nuclear receptor superfamily that regulate the gene expression of proteins involved in energy, glucose and lipid metabolism, adipocyte proliferation and differentiation and insulin sensitivity [1]. PPARs act as cellular sensors that activate transcription in response to the binding of natural or synthetic ligands. Three subtypes, PPARα, PPARβ/δ and PPARγ, have been identified. Although the subtypes share a high level of sequence and structural homology [2], they exhibit differences in tissue expression and physiological function [3]. Agonists of PPARα and PPARγ are currently approved for treating dyslipidemia and type 2 diabetes, respectively [4], [5]. Thiazolidinediones (TZDs) are one important class of synthetic agonists of PPARγ. TZDs are antidiabetic agents that target adipose tissue and improve insulin sensitivity, and they are currently being used in the treatment of type 2 diabetes. Despite the clinical benefit of TZDs, they have been associated with adverse side effects including weight gain, increased adipogenesis, renal fluid retention and a possible increased incidence of cardiovascular events [6]–[8]. Therefore, new PPARγ ligands with enhanced therapeutic efficacy and reduced adverse effects are needed. A promising new class of such ligands is that of the selective PPARγ modulators (i.e., SPPARγMs) [6]–[8]. These compounds act as partial agonists of PPARγ and display different binding properties than do full agonists [9]. The mechanism of PPARγ activation by full agonists is mediated by a molecular switch of the H12 α-helix [10]. H12 forms part of the ligand-dependent activation domain, AF-2, that closes on the ligand-binding site in response to ligand binding. The resulting active form can bind to several co-activator proteins that activate the cellular transcriptional machinery [10]. Full agonists occupy the large binding site of PPARγ in a U conformation and generally consist of a polar head and a hydrophobic tail [11]. The polar head makes a net of hydrogen bonds with the Ser289, His323, His449 and Tyr473 PPARγ side chains (Figure 1A), and this net is responsible for the conformational change of H12 and the activation of PPARγ [11]. In contrast, partial agonists activate PPARγ by an H12-independent mechanism [12], [13], and consequently, the key interactions between partial agonists and the ligand-binding domain (LBD) of PPARγ are different than those of the full agonists [9] (i.e., partial agonists do not bind to PPARγ by the net of hydrogen bonds used by full agonists). This causes a lower degree of H12 stabilization, which affects the recruitment of coactivators and, in turn, decreases the transcriptional activity of PPARγ [14], [15]. With minor exceptions, most of the currently known partial agonists interact with the LBD of PPARγ through a hydrogen bond with Ser342 [11] and several hydrophobic interactions that are similar to those that occur with full agonist binding (Figure 1B). Recently, a new mechanism by which partial and full PPARγ agonists act to improve insulin sensitivity independent of receptor agonism has been suggested. This mechanism consists of blocking the phosphorylation of PPARγ at Ser 273 [16] and may explain how partial agonists can exhibit similar or higher antidiabetic effects than those of full agonists. This mechanism might also be the reason for the differing side-effect profiles of the two types of agonists [8]. It is possible that partial and full agonists achieve comparable efficacy in insulin sensitization through a similar inhibitory effect on PPARγ phosphorylation, whereas the differences in their agonistic potency could be linked to the differences in side effects [8].

Although there are successful examples of the discovery of new PPARγ agonists [14], [17]–[20], including from natural origins [21]–[24], it has recently been of great interest to identify new PPARγ partial agonists from natural products [25], [26]. Consequently, the goal of this work was to design and apply a virtual screening (VS) workflow to identify novel PPARγ partial agonists among natural products. To achieve this goal, we **(a)** designed a VS workflow that includes a filter to remove PPARγ full agonist candidates from the sample; **(b)** validated the performance of the VS with samples of known PPARγ agonists (either full or partial) and decoys; **(c)** applied the VS to a database of natural or derivatives of natural compounds; **(d)** clustered the VS hits with known PPARγ partial agonists; and **(e)** selected 10 different VS hits (from 10 clusters where no known PPARγ partial agonists were present) for testing their bioactivity as PPARγ partial agonists. Our results show that our VS workflow performs well and is able to discover new chemical scaffolds for the design of effective antidiabetics with fewer side effects than PPARγ full agonists.

## Results and Discussion

### Virtual Screening: Description, Validation and Application

**Figure 2:**
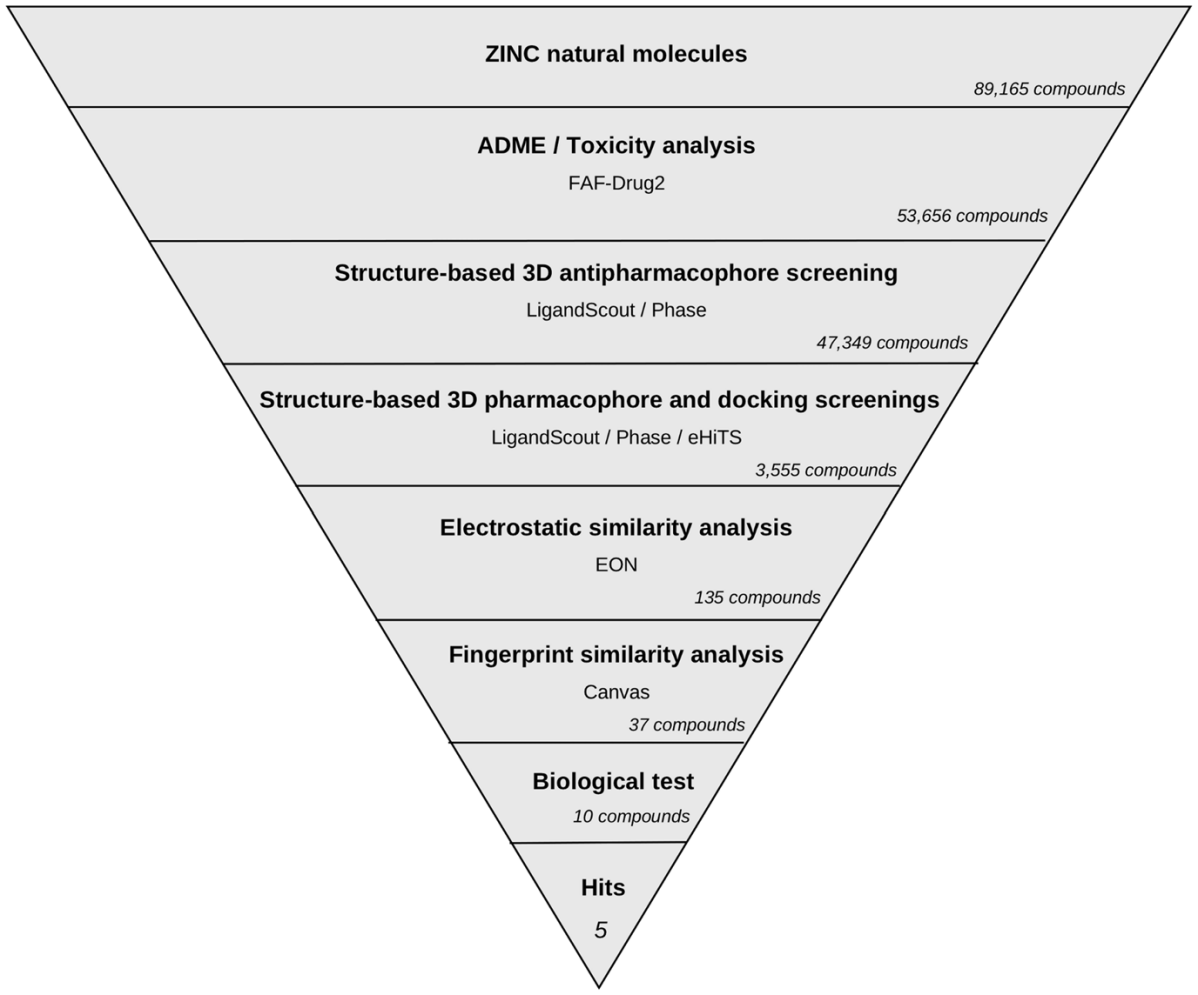
Schematic overview of the VS workflow and the procedure used for selecting the VS hits whose bioactivity was experimentally tested. The number of compounds that passed each step and the programs used are shown. From an initial set of 89,165 compounds, 135 compounds were identified as putative PPARγ partial agonists by the VS workflow. Ten of these 135 compounds were selected for *in vitro* testing.

**Figure 3:**
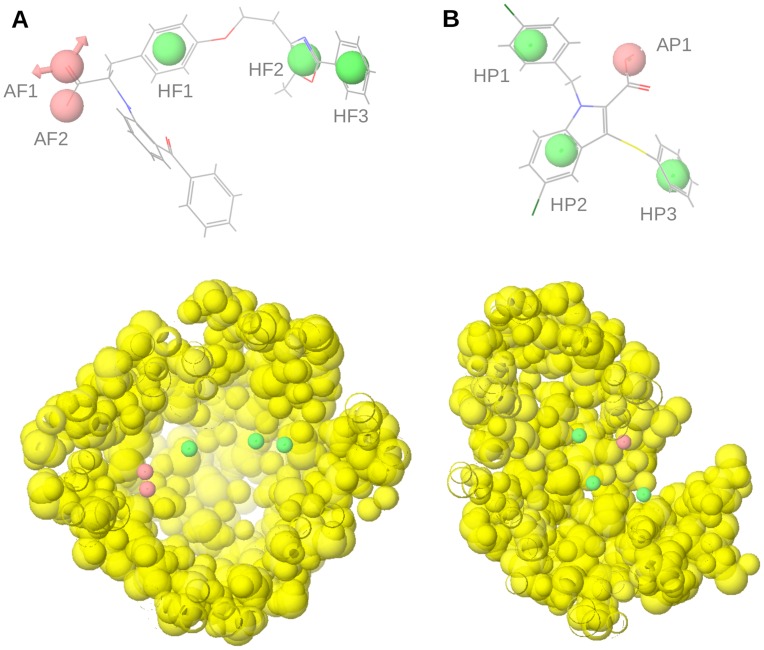
Pharmacophores used for the identification of (a) PPARγ full agonists and (B) PPARγ partial agonists. Hydrophobic and acceptor/donor sites are colored in green and pink, respectively. Excluded volumes are showed as yellow spheres. The ligands farglitazar (from the PDB entry 1FM9) and nTZDpa (from the PDB entry 2Q5S) are also represented.

The VS workflow applied in this study is summarized in Figure 2. It consists of several steps that were applied one after another (i.e., the output molecules of one step were the input molecules for the next step). The discriminatory power of the VS workflow to identify PPARγ partial agonists was evaluated by applying it to a group of 135 known PPARγ full agonists (Table S1), 19 known PPARγ partial agonists (Table S2) and all the decoys (i.e., [3,122]) available for PPARγ at the DUD database [27]. Table 1 shows how many of these molecules survived each VS step and several quantitative measures for model quality. Because we were interested in discovering novel PPARγ partial agonists but not full agonists, we first developed a structure-based pharmacophore, called the antipharmacophore, to exclude possible full agonists. We used this strategy because full agonists have more clearly defined structural features than partial agonists. Using 19 validated crystal structures from known full agonists complexed with PPARγ, we created the antipharmacophore model that represented the common features of full agonists. This antipharmacophore consisted of 5 sites (Figure 3A): 2 of them are involved in a hydrogen bond network between the ligand and the receptor, and 3 are hydrophobic sites. The PPARγ residues that interact with the two sites involved in the hydrogen bond network are Ser289, Tyr473, His323 and His449. Because the hydroxyl group from serine and tyrosine and the nitrogen from the histidine side chain can act as donors and acceptors simultaneously, the two sites involved in the hydrogen bond network were defined as having possible dual behavior as a hydrogen bond donor and acceptor. Both sites were considered to be essential. Two out of the three hydrophobic sites were also mandatory, whereas the site located at the effector end (site HF3 in Figure 3A) was defined as optional because it regulates the affinity and potency of ligands [11]. Table 1 shows that 104 out of the 135 PPARγ full agonists used in the validation process were identified as full agonists by our antipharmacophore model, as were 918 out of 3122 decoys (29%) and 7 out of the 19 partial agonists (37%). This represents an enrichment factor (EF) of 2.45, or 10% of the EF maximum value of 24.3 that would be obtained if all 135 full agonists were identified as positive hits in this analysis. Importantly, this is the only VS step for which the active set was composed of PPARγ full agonists and the inactive set was decoys and PPARγ partial agonists; therefore, the statistics for this VS step were calculated using these considerations. The sensitivity (Se) and the specificity (Sp) of this step were 77.04% and 29.45%, respectively. The high percentage of partial agonists misidentified as full agonists shows that it is sometimes difficult to distinguish between both sets. This was confirmed when some partial agonists were similar to full agonists and clustered together using a fingerprint similarity analysis (results not shown). However, because the aim of the antipharmacophore step was to minimize the presence of full agonists, loss of some possible partial agonists at this step was tolerated.

**Table 1:** Validation of each method used in the virtual screening workflow.

Set of Compounds	Number of Compounds	Structure-based pharmacophore screening			Electrostatic/shape similarity analysis	Global virtual screening
		anti pharmacophore	partial agonist pharmacophore			
		*in vacuo* *conformations*	*in vacuo* *conformations*	*docking* *poses*		
Partial Agonists	19	12	10	8	5	5
Full Agonists	135	31	11	7	1	1
Decoys	3122	2204	964	382	16	16
Enrichment Factor (EF)		2.45	1.90	1.98	11.28	39.19
EF_max_		24.27	187.25	98.50	49.63	172.42
Sensitivity (Se)		77.04%	83.33%	80.00%	62.50%	26.32%
Specificity (Sp)		29.45%	56.38%	60.10%	95.63%	99.49%

A dataset of 19 known PPARγ partial agonists, 135 known PPARγ full agonists and 3122 decoys extracted from the DUD database were used. The values represent the number of compounds from each set that survived each step when applied sequentially.

Next, a common PPARγ partial agonist pharmacophore, derived from 12 structures of PPARγ crystallized with a partial agonist, was applied. This pharmacophore consisted of one hydrogen bond acceptor, located on a carboxylic group of the ligand that forms a hydrogen bond with Ser342, and three hydrophobic sites located on aromatic rings (Figure 3B). From the 12 partial agonists that survived the antipharmacophore step, 10 were identified as partial agonists by our partial agonist pharmacophore, as were 11 of the 31 full agonists and 964 of the 2204 decoys. Therefore, the EF for this VS step was 1.90, which represents 1.02% of the maximum EF of 187.25 obtained if all 12 partial agonists would have been identified as true positives in this step. The Se and Sp for this step were 83.33% and 56.38%, respectively (Table 1).

To find docking poses that were compatible with the partial agonist pharmacophore, the compounds that had at least one conformer, generated *in vacuo,* that matched the partial agonist pharmacophore were also docked to the PPARγ structure from 2Q5S. The best docking poses were then matched again to the partial agonist pharmacophore. We found that 8 out of 10 partial agonists, 7 out of 11 full agonists and 382 out of 964 decoys that survived the previous step have at least one docked pose that both was compatible with the PPARγ ligand-binding site and had functional groups that match the 3D location of the sites of the partial agonist pharmacophore. The EF, Se and Sp for this step were 1.98, 80.00% and 60.10%, respectively (Table 1).

To reduce the number of PPARγ partial agonist candidates, an electrostatic and shape similarity analysis was applied. Using the experimental poses of 5 known PPARγ partial agonists as queries, 5 out of 8 partial agonists, 1 out of 7 full agonists and 16 out 382 decoys were identified as partial agonist candidates by this VS step. The EF, Se and Sp for this step were 11.28 (out of an EF maximum of 49.63), 62.50% and 95.63%, respectively (see Table 1). Although similar results are obtained without the docking process (data not shown), we prioritize obtaining docking poses to be safe that these matching conformations are biologically possible.

Table 1 shows that the full VS workflow identified 5 out of 19 partial agonists, 1 out of 135 full agonists and 16 out of 3122 decoys as partial agonists. Therefore, the global EF was 39.19 (22.73% of an EF maximum of 172.42) and the Se and Sp were 26.32% and 99.49%, respectively. The high Sp and the moderate Se of our procedure reflect, respectively, the correct assignment of inactive compounds and the loss of potential partial agonists. However, because of the high number of initial compounds and the difficulties in differentiating partial from full agonists, we preferred a very specific, but less sensible, VS workflow. Table 1 also shows that in terms of sensitivity, using the partial agonist pharmacophores was the best step, whereas in terms of specificity and EF, the best step was the electrostatic/shape similarity analysis. Therefore, the combination of the three steps seems adequate to obtain a VS workflow that combines the best of each method. Importantly, the Se and Sp of the antipharmacophore step should not be compared with those for the other VS steps because the objective of the antipharmacophore step was to remove full agonists from the sample. In that sense, despite the low Sp for this step, its high Se (77.04%) suggests that it is adequate for this purpose.

Upon validation of the VS workflow, it was applied to the Natural Products subset of the ZINC database [28]. From an initial set of 89,165 molecules, compounds with poor ADME properties or potentially toxic compounds were discarded, resulting in an initial set of 53,656 molecules. After applying the VS workflow described above, a group of 135 PPARγ partial agonist candidates were finally identified. Figure 2 shows the number of molecules that survived each step of the VS workflow.

### Fingerprint Similarity Analysis

To reduce the number of hits for biological testing while simultaneously increasing the significance of the results (i.e., by obtaining new chemical scaffolds for PPARγ partial agonists), a fingerprint cluster analysis was done. The 135 partial agonist candidates from the VS were combined with a group of 19 known partial agonists (Table S2), and their 2D fingerprints were calculated. A hierarchical cluster analysis classified the compounds into 51 clusters, and 37 of them did not contain any already known partial agonists and therefore represented new chemical scaffolds for PPARγ partial agonists.

### Biological Testing of Selected VS Hits

**Figure 4:**
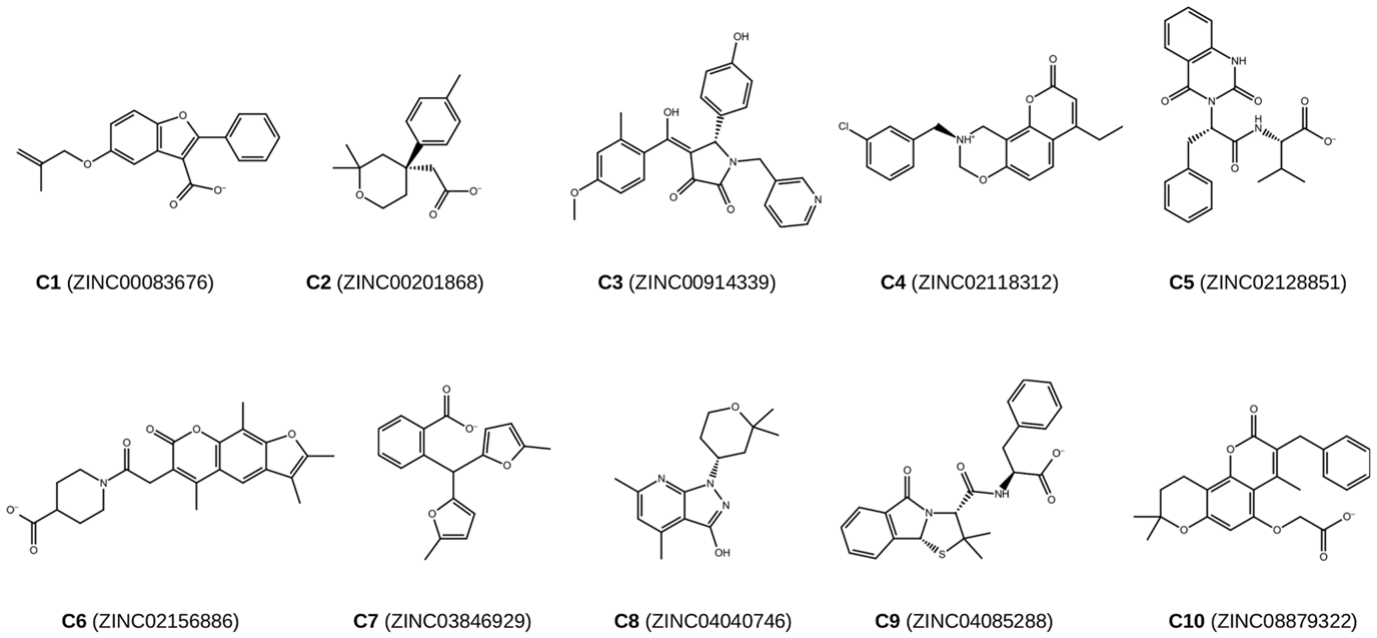
Chemical structures and ZINC codes of the 10 compounds suggested to be PPARγ partial agonists and selected for bioactivity testing.

Figure 4 shows the chemical structures of the ten compounds (C1-C10) selected for bioactivity testing. They were selected from 10 of the 37 clusters that corresponded to new chemical scaffolds of PPARγ partial agonists. The first selection criteria was to select the centroid molecule of each cluster, taking into account its price and availability. If the centroid molecule was not available, we also took into account the value of the ET_combo score, prioritizing the highest values. We also prioritized the most pure and cheapest compounds and obtaining all the compounds from the same vendor. To avoid any bias in the selection process and discover new PPARγ partial agonists, we checked that the PPAR-related activity of the selected compounds were not known.

It is likely that at least some of the problematic side effects of PPARγ full agonists, such as weight gain or fluid retention, may be caused by classical agonist interactions. A substantial portion of the therapeutic benefits of full and partial PPARγ agonists occurs through the inhibition of the PPARγ phosphorylation at Ser273 [16]. Thus, an effective partial agonist of PPARγ would have weak or low transactivation activity while maintaining the stimulation of glucose uptake [16]. In this sense, the ten selected compounds were analyzed *in vitro* to check whether they bind to PPARγ, activate the transactivation activity of PPARγ, and stimulate glucose uptake and differentiation in adipocytes. Prior to these analyses, we performed cytotoxicity and viability tests in HepG2 and 3T3-L1 cells. Only C1, C8 and C9 at 1000 μm show a significant decreased of viability in 3T3-L1 cells.

**Figure 5:**
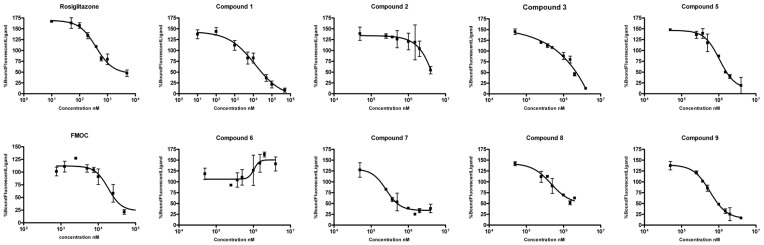
Results of the PolarScreen PPAR Competitor Assay for all selected compounds, except C4 and C10, which had solubility problems. One PPARγ full agonist (Rosiglitazone) and one partial agonist (FMOC) were also assayed. Error bars represent one standard deviation from the mean of triplicates.

Figure 5 shows the results of the PolarScreen PPARγ Competitor Assay to determine the binding affinity of the selected compounds. Compounds C4 and C10 were not assayed due to solubility problems. C10 has an estimated log P value of 4.3 (the highest value between the 10 compounds) and C4 has an estimated log P value of 3.5 (the third highest value between the 10 compounds). These indicate that these compounds have a less polarity than the rest of the compounds and that it could be implemented a log P prediction step (during the ADME/Toxicity prediction step) for not selecting low solubility compounds. Compounds C1, C5, C7, C8 and C9 bound to PPARγ with different affinities (Table 2). C1 had a moderate binding affinity for PPARγ, similar to that of the known PPARγ partial agonist FMOC. Compounds C5, C7, C8 and C9 had lower binding affinities. The results for compounds C2, C3 and C6 were not conclusive when assayed at concentrations up to 8 mM. These results validate the predictions of the VS procedure, as five out of eight of the assayed compounds were able to bind PPARγ. Table 2 also shows the transcription activity of the five compounds that were able to bind PPARγ and were potential PPARγ partial agonists (C1, C5, C7, C8 and C9). Only compounds C1 (at 100, 500 and 1000 μM) and C7 (at 1000 μM) have a moderate and significant transactivation activity, but lower than Rosiglitazone and similar or lower to that shown by the partial agonist FMOC (see Table 2). These results validate the antipharmacophore step, as none of the assayed compounds can be considered as a PPARγ full agonist.

**Table 2:** Experimental IC_50_ values and transactivation activity of the selected compounds.

		Gene Reporter activity at			
Compound	Binding affinityIC_50_ (μM)	10 μMmean ± SD	100 μMmean ± SD	500 μMmean ± SD	1000 μMmean ± SD
Rosiglitazone	0.39	5.421±0.889**	10.443±3.798**		
FMOC	18.5	2.153±0.176	3.171±0.133^*^		
C1	12.4	1.015±0.147	5.669±0.668**	8.126±1.233**	9.701±1.193**
C5	1000	1.129±0.773	0.906±0.061	0.962±0.043	0.979±0.149
C7	252	1.951±1.368	1.365±0.189	1.527±0.283	3.579±0.789**
C8	460	0.631±0.166	0.864±0.185	0.970±0.165	1.210±0.186
C9	585	1.192±0.346	0.939±0.138	1.103±0.082	1.423±0.203

A competitive binding assay was used to assess the ability of experimental compounds, FMOC or rosiglitazone to displace a fluorescent PPARγ ligand from a human-derived recombinant PPARγ ligand-binding domain. The concentration of the test compound that results in a half-maximal shift in the polarization value is defined as IC_50_. This value is a measure of the relative affinity of the test compound for the PPAR ligand-binding domain. The transactivation capacity of selected compounds was also determined in HepG2 cells as described in Materials and Methods. Results represent the mean ± SD of at least three separate experiments performed in triplicate. Results are expressed as arbitrary firefly luciferase units relative to arbitrary renilla luciferase units.

For compounds C2, C3 and C6, no binding was observed when assayed at concentrations up to 8 mM. Compounds C4 and C10 were not assayed because of solubility problems. For Rosiglitazone and FMOC, gene reporter activity at 500 and 1000 μM were not assayed because of solubility problems.

** p<0.001 and * p<0.05 when compared to the control (DMSO) in an ANOVA test.

The *in vitro* effects of these five partial agonists (C1, C5, C7, C8 and C9) on the adipogenic activity are shown in Table 3. As expected from the results of the PPARγ transactivation activity assay, none of the assayed compounds induced triglyceride accumulation in 3T3-L1 preadipocytes at 100 μM and 1000 μM concentrations. Table 4 shows the results of the effects on insulin-induced glucose uptake in adipocytes. All of the selected compounds stimulated insulin-induced glucose uptake to the same extent or even more than Rosiglitazone and FMOC, with compounds C7, C8, and especially C5 being the most effective. Together, these results show that these five compounds can be considered to be partial agonists of PPARγ and validate the virtual screening protocol developed.

**Table 3:** In vitro assay of adipogenic activity of some of the selected compounds.

	µg TG/well	
**vehicle**	2,473±0,06	
**Control differentiation**	18,668±2,49 #	
Dose of compound	**100 µM**	**1000 µM**
C1	2,999±0,38	
C5	2,644±0,19	2,543±0,35
C7	2,483±0,24	1,656±0,19 *
C8	1,791±0,32	
C9	2,403±0,31	

The compounds that bind PPARγ were added to 3T3-L1 pre-adipocytes to test their adipogenic capacity, measured as triglyceride accumulation. All the compounds were tested at the non-toxic 100 µM dose. A higher (1000 µM) dose was assayed for those compounds in which it did not exert citotoxic effects. A control of differentiation was performed by inducing cells to differentiate with an hormonal cocktail (insulin, IBMX, dexamethasone) as described in matherials and methods. Data are mean ± SEM of 3–6 biological replicates.

# p≤0.01 vs vehicle; * p≤0.05 vs vehicle; all results significantly different (p≤0.01 ) than the control of differentiation.

**Table 4:** Stimulation of glucose uptake by some of the selected compounds measured *in vitro*.

Compound	Glucose uptake stimulation
Rosiglitazone	118.99±8.543*
FMOC	124.64±7.295*
C1	120.9±13.561**
C5	140.48±17.385*
C7	133.54±13.508*
C8	120.24±6.680*
C9	120.90±20.410

The compounds that bind PPARγ were added to fully differentiated adipocytes to test their effects in inspone.0050816.g007.tifulin-stimulated 2-deoxy-[H^3^]-glucose uptake. Values are shown as % of insulin stimulation (insulin stimulation is considered 100%). Data are mean ± SEM of at least three biological replicates.

* p<0.05 ** p<0.1 vs. insulin.

### Docking of Novel PPARγ Ligands

**Figure 6:**
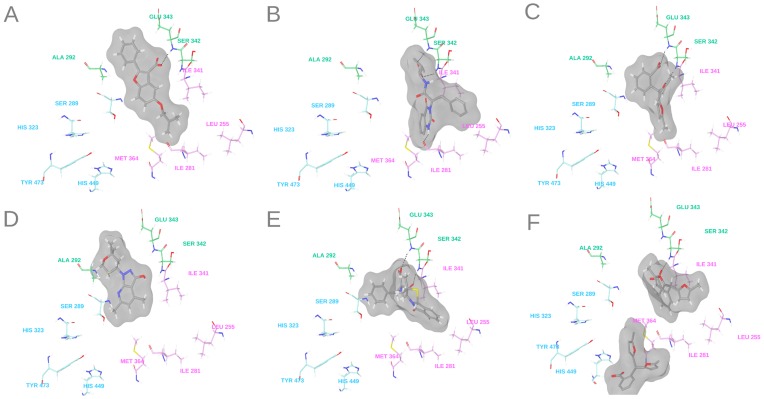
Putative ligand-PPARγ interactions of the best docking poses of compounds (A) C1, (B) C5, (C) and (F) C7, (D) C8 and (E) C9. The following key residues of the LBD of PPARγ are shown: Ser289, His 323, Tyr473 and His449 from arm I are colored in blue; Leu255, Ile281, Ile341 and Met364 from arm II are colored in pink; and Ala292, Ser342 and Glu343 from arm III are colored in green.

To determine the putative binding mode and the potential ligand-target interactions of the five novel PPARγ partial agonists (C1, C5, C7, C8 and C9), these compounds were docked to the PPARγ LBD of PDB entry 2Q5S. Similar docking poses were determined for all sets of compounds (see Figure 6). The predicted binding modes of all compounds (with the exception of C8) included one hydrogen bond with Ser342 or adjacent residues and several hydrophobic contacts with Ile281, Ala292, Ile326, Ile341, Leu330, Leu333, Val339, Met348, Leu353 or Met364 from arms II and III of the LBD of PPARγ. These interactions are typical of PPARγ partial agonists [9]. In addition, no hydrogen bond interaction between the five compounds and residues His323, Tyr327, His449 and Tyr473 from arm I of the LBD of PPARγ (typical of PPARγ full agonists) was predicted. This could explain the lack of (or moderate) transactivation activity determined for the five compounds.

The best docking pose for compound C5 shows that this compound could establish three hydrogen bonds with arms II and III of the LBD of PPARγ (Figure 6B). Two of these hydrogen bonds include those formed between the carboxylic moiety of the compound and the backbones of Ser342 and Glu343. Another hydrogen bond could be established between the nitrogen of the quinazoline-2,4-dione moiety and the side chain of Ile281 (Figure 6B). In addition, hydrophobic interactions were predicted between the rest of the ligand and residues Ile249, Leu255, Arg288, Met348, Val339, Ile341, and Met364 from arms II and III. The initial docking of compound C7 to the LBD of PPARγ showed that this compound could establish two hydrogen bonds with Ser342 and Glu343 (Figure 6C). The small size of this compound may allow the binding of a second ligand molecule. This 2∶1 binding stoichiometry has been described or predicted for other PPARγ partial agonists [25], [29]. Based on this, we performed a docking study to investigate the possibility that two copies of compound C7 could bind simultaneously to PPARγ. Figure 6F shows that an additional copy of the C7 compound could interact with arms I and III through several hydrophobic interactions. The experimental IC_50_ and transactivation activity of this compound also suggest this possibility. The plot of the PolarScreen PPAR competitor assay for compound C7 in Figure 5 shows that the full binding is delayed until high concentrations are reached. In the same way, we did not observe significant transactivation activity with 10 μM of this compound, but this activity increases significantly with 100 μM (see Table 2). These observations agree with a model in which one molecule of compound C7 binds to arms II and III of the LBD of PPARγ, and when the concentration increases, a second molecule occupies arm I and causes transactivation activity.

### Conclusions

We have shown that a VS workflow based on two structure-based 3D pharmacophores (one to exclude potential PPARγ full agonists), protein-ligand docking and electrostatic/shape similarity analysis is able to discover novel scaffolds for PPARγ partial agonists. Thus, from an initial set of 89,165 natural products and natural product derivatives, 135 compounds were defined as potential PPARγ partial agonists. Using a fingerprint similarity analysis, 37 clusters that represent new chemical scaffolds for PPARγ partial agonists were defined. Ten compounds from ten of these clusters were chosen for bioactivity testing, but two of them were not assayed because of solubility problems. Five out of the remaining eight compounds can be considered as PPARγ partial agonists because they were able to bind PPARγ with a moderate affinity, did not stimulate adipogenesis and enhanced insulin-stimulated glucose uptake *in vitro*. Therefore, our results suggest that our VS workflow is able to identify compounds with a high chance of being effective PPARγ partial agonists in a molecule database and that this bioactivity is not trivial because their chemical structure does not resemble known PPARγ partial agonists. In addition, our data show that C5 is an appropriate compound for lead-optimization and the subsequent design of more potent and safe antidiabetic drugs.

## Materials and Methods

### Dataset of PPARγ Structures Used

Forty-nine structures of the PPARγ LBD co-crystallized with an agonist were downloaded from the RCBS Protein Data Bank (http://www.pdb.org) [30]. For each structure, we determined whether electron density maps were available at the Uppsala Electron Density Server (http://eds.bmc.uu.se/eds/) [31] and, if available, the goodness-of-fit between these maps and the structures of both the ligand and the PPARγ active site. After this preliminary analysis, 18 out of the 49 PDB complexes were not further considered in our study due to one of following reasons: **(a)** the electron density maps were not available; **(b)** either the ligand or the PPARγ active site did not fit well on the electron density maps; **(c)** the ligand was a fatty acid; or **(d)** the ligand could not be identified as either a full or partial agonist. The remaining 31 PDB complexes (Table 5) were superposed with the DeepView v3.7 program [32] to arrange them in the same relative orientation. Only the resulting re-oriented coordinates of the PDB complexes were used in the subsequent steps of the workflow.

**Table 5:** PDB codes of the ligand-protein complexes used for the generation of the structure-based pharmacophore models for PPARγ full agonists and PPARγ partial agonists.

Full agonists				Partial agonists				
				cluster 1	cluster 2	cluster 3	cluster 4	cluster 5
1FM9	1I7I	1FM6	2GTK	2G0G	4PRG	2Q6R	2FVJ	2Q6S
1RDT	1KNU	1ZGY	3B3K	2G0H		2Q61		2WM0
1K74	2F4B	2PRG	2ATH			2Q5S		
3BC5	2HWQ	2FVJ	1NYX			2Q5P		
2Q8S	2HWR	1ZEO				2HFP		
						2P4Y		

### Generation of Structure-Based Pharmacophores

LigandScout v2.03 (Inte:ligand, Vienna, Austria, http://www.inteligand.com/ligandscout/) [33], [34] was used for the analysis of the 31 PPARγ structures from Table 5 and the analysis of the possible interactions between the crystallized ligands and the ligand-binding pocket of PPARγ. Individual pharmacophores for the 19 structures of PPARγ crystallized with a full agonist (Table 5) were visually inspected to construct a common structure-based pharmacophore of full agonists. This pharmacophore (Figure 3A) is formed by 5 sites (two hydrogen-bond acceptors and three hydrophobic sites) that are present in most of the complexes of full agonists analyzed and are therefore assumed to be responsible for the intermolecular interactions that are essential for the activity of PPARγ full agonists. We named this pharmacophore the antipharmacophore because we used it to exclude putative full agonists when searching for partial agonists. Taking into account ligand similarity, we classified the remaining 12 structures of PPARγ crystallized with a partial agonist into 5 clusters or families (Table 5). For each cluster, a common structure-based pharmacophore for PPARγ partial agonists was defined. The resulting pharmacophores contained 5 to 8 sites, mainly hydrophobic sites and some hydrogen-bond acceptors. A common pharmacophore of 4 sites was then constructed. This pharmacophore (Figure 3B) consisted of one hydrogen-bond acceptor site (site AP1 at Figure3B) that interacts with Ser342 and three hydrophobic sites (sites HP1, HP2, HP3 at Figure 3B) that make hydrophobic interactions with residues from arm II (*i.e.* Ile281, Val339, Ile341, Met348, Leu353 and Met364) and arm III (*i.e.* Ala292 and Leu333). This common pharmacophore contained the sites in common with the pharmacophores of each cluster and, in our opinion, the sites that are important for the intermolecular interaction between PPARγ and its partial agonists. This common pharmacophore, which we called the partial agonist pharmacophore, was used in the VS workflow to identify putative PPARγ partial agonists.

Both pharmacophores were also completed with receptor-based excluded volumes, obtained either from 1FM9 (that contains the full agonist Farglitazar) for the antipharmacophore or from 2Q5S (that contains the partial agonist nTZDpa) for the partial agonist pharmacophore, that schematically represent the location of the PPARγ residues that form the LBD (Figures 3C and 3D). Other validated structures could be used, but the superposition of all the available structures containing a PPARγ full or partial agonist suggests that similar results would be obtained. Excluded volumes were added by applying the Receptor-Based Excluded Volumes graphic front-end from Phase v3.1 (Schrödinger LLC., Portland, USA; http://www.schrodinger.com) [35] and by setting the Sphere filter parameter values to **(**a**)** ignoring receptor atoms whose surfaces were within 0.25 Å of ligand surface and **(**b**)** limiting excluded volume shell thickness to 10 Å. The rest of the parameters used were the default values.

### Initial Dataset of Natural Compounds

The initial dataset of the natural compounds that we used contained 89,165 compounds from the Natural Products subset of the ZINC database (http://wiki.compbio.ucsf.edu/wiki/index.php/Natural_products_database) [28]. We used the ZINC database because it contains commercially-available natural products and natural-product derivatives. Thus, we could purchase and test *in vitro* the bioactivity of the selected compounds. We will assess in a separate manuscript the identification of some natural extracts that contain a predicted PPARγ partial agonists by our virtual screening procedure. The 3D structures of this initial dataset were processed with the LigPrep v2.3 program (Schrödinger LLC., Portland, USA; http://www.schrodinger.com) to clean them and obtain their corresponding low-energy structures. Only one low energy conformation was generated for each molecule. This process was carried out with the following parameter values: (a) the force field used was OPLS 2005; (b) all possible ionization states at pH 7.0±2.0 were generated with Ionizer; (c) the desalt option was activated; (d) tautomers were generated for all ionization states at pH 7.0±2.0; (e) chiralities, when present, were determined from the 3D structure; and (f) one low-energy ring conformation per ligand was generated. When chirality was not defined, a maximum number of 32 stereoisomers were generated. Conformations were built with the Phase program, generating *in vacuo* a maximum number of 200 conformers per structure and using the default Phase options.

### Virtual Screening Workflow

Briefly, the VS workflow consisted of several steps that must be applied one after another (i.e., the output molecules of one step were the input molecules for the next step). Thus, the filters applied and sorted according their usage were: (1) an ADME/Toxicity prediction; (2) a structure-based antipharmacophore screening for removing PPARγ full agonist candidates; (3) a structure-based pharmacophore screening; and (4) an electrostatic/shape similarity analysis.

The initial set of compounds was submitted to an ADME/Tox filter with the FAF-Drugs2 program [36]. The aim of this step was to discard those molecules that could have poor ADME properties or were potentially toxic. Thus, the drug-like properties of a compound were evaluated by means of the Lipinski rule [37]_,_ and only one violation of the rule was allowed. This rule is based on a set of property values (i.e., the number of hydrogen bond donors and acceptors, the molecular weight and the logP) that were derived from a large number of drugs with good ADME characteristics [37]. Hence, molecules that pass the Lipinski rule are expected to be orally active in humans. Moreover, molecules containing toxic groups were filtered by using the 204 substructures for “warhead” chelators, frequent hitters, promiscuous inhibitors and other undesirable functional groups available in the FAF-Drugs2 [36] tool.

Molecules with appropriate ADME/Tox properties were then filtered by a structure-based antipharmacophore with the aim of discarding potential PPARγ full agonists. This filter removed from the sample those molecules that had at least one *in vacuo*-generated conformer that matched at least 4 out of 5 sites of the antipharmacophore. The fitting between the molecules and the pharmacophore was analyzed with the Phase program [33], using a site-matching tolerance of 2 Å for acceptor and donor sites and 2.5 Å for hydrophobic, aromatic and negative sites and applying the excluded volumes previously generated. The subset of molecules that did not match the antipharmacophore was then used to identify possible partial agonists. To do this, a second pharmacophore obtained from the common sites of known PPARγ partial agonists was used. Equivalent conditions were used for the pharmacophore-based searches. Molecules that had at least one *in vacuo*-generated conformer and matched the 4 sites of the partial agonist pharmacophore were initially identified as putative PPARγ partial agonists.

To find docking poses that were compatible with the partial agonist pharmacophore, the molecules identified as putative PPARγ partial agonists were docked to the ligand-binding site of 2Q5S. The 32 best docked poses predicted by the eHiTS v2009 program (SimBioSys Inc., Toronto, Canada; http://www.simbiosys.ca/ehits) [38] were filtered again with Phase through the partial agonist pharmacophore, using the same filtering options of the first pharmacophore matching, with the exception that now re-orientation of the poses was not allowed during the search (i.e., the score in place option was used). The reason for using eHiTS was basically the available computational time, because at this step we still had a high number of candidates to become PPARγ partial agonists. Latter, for the last selected molecules, we did an exhaustive docking with Glide XP that also provides additional descriptors which helped us to analyze and visualize the docking poses in a comprehensive way. Both softwares provide reasonable poses that contain known interactions with PPARγ, following the same binding mode.

The poses that passed the pharmacophore and docking screenings were submitted to an electrostatic/shape similarity analysis, using the experimental poses of the PPARγ partial agonists crystallized at the structures 2G0H, 4PRG, 2Q5S, 2FVJ and 2Q6S as queries. These five partial agonists were selected from each of the five clusters of PPARγ partial agonists defined in Table 5. Cluster 2 and 4 contain only one structure and we chose the ligands present in these structures. For cluster 3 we choose the 2Q5S structure because this was the structure chosen for adding the receptor-based excluded volumes at the partial agonist pharmacophore. Cluster 1 and 5 contain only two molecules, and we chose one of them at random. The electrostatic/shape similarity analysis was done with EON v2.0.1 (OpenEye Scientific Software, Inc., Santa Fe, New Mexico, USA; http://www.eyesopen.com) using the Electrostatic Tanimoto combo (ET_combo) score as similarity criteria. We applied the default conditions, using the Molecular Force Field MMFF94, in order to calculate the partial charges of the molecules. The ET_combo score is the sum of two calculations: the Shape Tanimoto (*ST*) score, which is a quantitative measure of three-dimensional overlap, where 1 corresponds to a perfect overlap, i.e., same shape, and the Possion-Boltzman Electrostatic Tanimoto (ET_pb) score that compares the electrostatic potential of two small molecules and ranges from 1 (identical potential) to negative values that result from the overlap of positive and negative charges. Molecules with an ET_pb score greater than 0.3 and an ST score greater than 0.5 were predicted to be potential PPARγ partial agonists by the VS workflow.

### Virtual Screening Workflow Validation

The ability of the VS workflow to identify PPARγ partial agonists was tested by applying it to a group of 135 known PPARγ full agonists (Table S1), 19 known PPARγ partial agonists (Table S2) and 3,122 decoys obtained from the DUD database [27]. The structures of the 135 full agonists and the 19 partial agonists were built with ChemDraw Ultra v11.0 (CambridgeSoft Corporation, Cambridge, MA, USA; http://www.cambridgesoft.com/) [39] and cleaned using LigPrep v2.3 (Schrödinger LLC., Portland, USA; http://www.schrodinger.com). For each step of the VS workflow, an enrichment factor (EF) and a value for sensitivity (Se) and specificity (Sp) were calculated [40]. The EF was defined as the quotient of the fraction of active compounds in the sample that survived a particular VS step and the fraction of active compounds that were in the sample before applying this step. Therefore, the EF represents the ratio of the number of active compounds actually retrieved by a method compared with the number expected purely by chance. The maximum EF value (EF_max_) at each step was also estimated assuming only the known active compounds would survive at each step. Sensitivity (Se) describes how well the model correctly identifies active compounds, and it is calculated as the ratio between the number of active compounds that survived a particular VS step and the number of all active compounds that were in the sample before applying the VS step. Specificity (Sp) measures the correct assignment of inactive compounds, and it is calculated as the ratio between the number of inactive compounds that were discarded at a particular VS step and the number of all inactive molecules that were in the sample before applying the VS step. For the estimation of EF, EF_max_, Se and Sp at the antipharmacophore step, the full agonist set was considered to be the set of active compounds. For the rest of the steps, the set of partial agonists was considered to be the active compounds. Global EF, EF_max_, Se and Sp values for the entire VS process were also calculated using the number of active or inactive compounds that survived the entire VS workflow and the initial number of compounds before applying the VS procedure.

### Structural Similarity Analysis

To select a representative dataset of VS hits for testing their bioactivity, the molecules that survived the electrostatic/shape similarity filter were merged with a group of 19 known partial agonists (Table S2) and clustered with Canvas v1.2 (Schrödinger LLC., Portland, USA; http://www.schrodinger.com). MOLPRINT2D fingerprints [41] with a fingerprint precision of 32 bits were calculated for each molecule, and then a hierarchical clustering based on Tanimoto similarities was obtained. The number of clusters was defined using the Kelley criterion [42]. Clusters that did not contain any known partial agonists were defined as clusters with new scaffolds for PPARγ partial agonists, and ten molecules from ten different clusters were selected for further bioactivity tests.

### Reagents and Materials for the Biological Tests

The 10 selected compounds [ZINC00083676 (C1), ZINC00201868 (C2), ZINC00914339 (C3), ZINC02118312 (C4), ZINC02128851 (C5), ZINC02156886 (C6), ZINC03846929 (C7), ZINC04040746 (C8), ZINC04085288 (C9), and ZINC08879322 (C10)] were purchased from InterBioScreen Ltd. (Moscow, Russia). Their purities were higher than 92% or 95%. FMOC-L-Leucine (FMOC) was purchased from Calbiochem (Merck, Darmstadt, Germany). Rosiglitazone (BRL) was kindly provided by GlaxoSmithKline (Middlesex, UK). The test compounds were dissolved in DMSO, aliquoted and kept frozen until use. Cell culture reagents were obtained from BioWhittaker (Verviers, Belgium). Bradford protein reagent was obtained from Bio-Rad Laboratories (Life Sciences Group, Hercules, CA, USA). Insulin (Actrapid) was from Novo Nordisk (Bagsvaerd, Denmark). 2-deoxy-[H3]-glucose and ECL detection reagent were from Amersham Biosciences (Buckinghamshire, England).

### Polarscreen PPARγ Competitive Assay

The PPAR ligand-binding competitive assay was performed with the PolarScreen™ PPARγ Competitor Assay Green according to the manufacturer’s protocol. Briefly, the PPARγ LBD and the fluorescent PPARγ ligand form a complex with a high polarization value. Displacement of the fluorescent ligand by PPARγ ligands frees the fluormone in solution to tumble rapidly during its fluorescence lifetime, causing a low polarization value. The change in polarization value was used to determine the relative affinity of test compounds for the PPARγ LBD. Fluorescence polarization was measured using a POLARstar omega plate reader (BMG Labtech, Germany) at an excitation wavelength of 485 nm and an emission wavelength of 535 nm. Rosiglitazone, a compound with high affinity for PPARγ, was used as a positive control. Polarization values were plotted against the concentration of the test compound. To discard non-specific effects, DMSO was also tested at equivalent concentrations. The concentration of the test compound that resulted in a half-maximal shift in polarization value was defined as IC_50_. This value is a measure of the relative affinity of the test compound for the PPAR LBD. Curve fitting was performed using GraphPad Prism v4.0 (GraphPad Software, San Diego CA, USA; http://www.graphpad.com) following the program instructions.

### Dual-Luciferase Reporter Assay

The activity of overexpressed PPARγ in response to its agonists was assessed in HepG2 cells (purchased from the European Collection of Cell Cultures) using a PPARγ reporter (SABiosciences CCS-3026L). The PPAR reporter is a mixture of a PPAR-responsive luciferase construct and a constitutively expressed Renilla construct (40∶1). The PPAR-responsive luciferase construct encodes the firefly luciferase reporter gene under the control of a minimal (m)CMV promoter and tandem repeats of the PPAR transcriptional response element. This construct monitors both increases and decreases in the transcriptional activity of PPAR. The constitutively expressed Renilla construct encodes the Renilla luciferase reporter gene under the control of a CMV immediately early enhancer/promoter and acts as an internal control for normalizing transfection efficiency and monitoring cell viability. Cells were co-transfected with the PPAR reporter and negative control along with the PPARγ expression vector in a 96-well plate. After 24 hours of transfection, cells were treated with the total agonist rosiglitazone (1 μM), partial agonist FMOC (10 μM) or the selected experimental compounds (10 and 100 μM). The dual-luciferase assay was performed with the Biotek FLx800 Multi-Detection Microplate Reader using the Promega dual luciferase reporter kit (E1910). Promoter activity values are expressed as arbitrary units using a Renilla reporter for internal normalization. Experiments were done in at least triplicate, and results represent the relative luciferase activity normalized to the untreated control. Statistical analysis was carried out by one-way analysis of variance (ANOVA) with Dunnett’s post-hoc test using GraphPad Prism v4.0. Differences were considered significant when P<0.05* or P<0.001**.

### Cytotoxicity and Viability Assays of the Experimental Compounds in HepG2 Cells

HepG2 cytotoxicity induced by the tested compounds was assessed by lactate dehydrogenase (LDH) leakage into the culture medium. Following a 24-h exposure to compounds C1–C10 (10 and 100 μM), the culture medium was aspirated and centrifuged at 3000 rpm for 5 min to obtain a cell-free supernatant. The activity of LDH in the medium was determined using a commercially available kit from QCA (Amposta, Spain). Aliquots of media and warm reagent were mixed in a 96-well plate (Falcon, 353075), and the decrease in absorbance was recorded using a microplate spectrophotometer system (Biochrom, UK). Results were analyzed with GraphPad Prism v4.0 and presented as LDH activity (mU/ml).

An MTT test was used to assess viability. HepG2 cells, cultured at a density of 5.0 × 10^4^ in a 96-well plate in Dulbecco’s modified Eagle’s medium (DMEM), were treated with compounds C1–C10 (10 and 100 μM) for 24 hours. After the medium was changed, HepG2 cells were treated with 5 mg/ml MTT (Thiazolyl Blue Tetrazolium Bromide) solution (Sigma, M5655) for 4 hours. After cells were dissolved in DMSO, the level of formazane was analyzed by measuring the optical density at 570 nm against the optical density at 630 nm. Results were analyzed with GraphPad Prism v4.0 and are presented as the percent viability of control values.

### 3T3-L1 Preadipocyte Cell Culture and Treatment

The 3T3-L1 preadipocyte cell line (purchased from the American Type Culture Collection) was used to evaluate the adipogenic activity and the stimulation of the insulin-induced glucose uptake of selected compounds. 3T3-L1 pre-adipocytes were propagated in 24-well plates and induced to differentiate in DMEM. Proliferating preadipocytes were maintained at low density in a culture medium (growth medium) that consisted of DMEM supplemented with 10% calf serum, 2 mM glutamine, 100 U/ml penicillin and 100 µg/ml streptomycin. For the differentiation assay, 2-days post-confluent preadipocytes were treated with 200 nM insulin and different doses (100 µM and 1 mM) of test compound for 6 days in DMEM supplemented with 10% fetal bovine serum (FBS). The treatment medium was changed every 2 days. Then, toxicity and triglyceride content were measured. Controls of differentiation were performed treating the cells with a differentiation cocktail as previously described [43]. Cellular viability was assessed by the neutral red assay [44]. For the glucose uptake assay, cells were differentiated with the previously described differentiation cocktail [43]. Briefly, cells were treated with 0.25 µM dexamethasone, 0.5 mM 3-isobutyl-methylxanthine, and 200 nM insulin for 2 days in DMEM containing 10% FBS, then switched to the same media containing insulin for 2 more days, and then switched to the same media without insulin. Ten days after differentiation was induced, cells were treated with the test compounds (1 µM) for 3 more days and used for the glucose uptake assay.

### Evaluation of the Adipogenic Activity of the Selected Compounds

Treated cells were rinsed twice with PBS, scraped into a 250-µl solution of 50 mM Tris-HCl, 1 mM EDTA and 1 mM b-mercaptoethanol and sonicated. The resulting cell lysates were used to determine the total triacylglyceride content, measured using the enzymatic glycerol-phosphate oxidase test, following the manufacturer’s instructions (QCA, Amposta, Spain). Results were expressed as the mean ± SEM. The effects were assessed using a one-way ANOVA or Student’s T-test. We used Tukey’s Test of honestly significant differences to make pairwise comparisons. All calculations were performed using SPSS (IBM Corp., New York, USA).

### Glucose Uptake Assay

After the treatment of 3T3-L1 adipocytes with the different compounds, the cells were serum-depleted for 3 hours, and 200 nM insulin or water (vehicle control) was added for 30 min. Glucose transport was determined by measuring the 2-deoxy-d-[^3^H]glucose uptake as previously described [45]. Protein content assessed by the Bradford method [46] was used to normalize the glucose transport values. Each condition was run in triplicate. Results were expressed as the mean ± SEM. The effects were assessed using a one-way ANOVA or Student’s T-test. We used Tukey’s Test of honestly significant differences to make pairwise comparisons. All calculations were performed using SPSS.

### Docking of Novel PPARγ Partial Agonists

Docking studies of the PPARγ partial agonists C1, C5, C7, C8 and C9 were performed with the software Glide v5.6 (Schrödinger LLC., Portland, USA; http://www.schrodinger.com) on the PPARγ crystal structure 2Q5S. For compound C7, an additional docking study was performed with the 2HFP structure. This structure was chosen because in this structure two molecules of the same ligand were seen to span the binding pocket of PPARγ showing a 2∶1 stoichiometry of binding [29]. The binding site was defined using the Receptor Grid Generation panel with the default options. Extra-precision (XP) docking was selected for screening the ligands. We selected the flexible docking mode, meaning that Glide internally generated the conformations during the docking process. We did not request any constraints for docking. Each docking run recorded a maximum of ten poses per ligand that survived the post-docking minimization. GlideScore XP was used as the fitness function. The best docking poses for the novel PPARγ ligands were selected by taking into account not only the docking scores but also the results of the visual investigation of all docking poses. Maestro v9.2 and Glide XP Visualizer (Schrödinger LLC., Portland, USA; http://www.schrodinger.com) were used for analyzing and visually investigating the ligand-protein interactions of the docking poses.

## Supporting Information

Table S1 **Structures of the 135 PPARγ full agonists used in the VS validation.**
(PDF)

Table S2 **Structures of the 19 PPARγ partial agonists used in the VS validation.**
(PDF)
