# Supplementary material for: Identification of PPARgamma Partial Agonists of Natural Origin (I): Development of a Virtual Screening Procedure and In Vitro Validation
Source: PLoS One. 2012 Nov 30;7(11):e50816. doi: 10.1371/journal.pone.0050816 (PMC3511273; doi:10.1371/journal.pone.0050816)
Supplement: Table S1 — Structures of the 135 PPARγ full agonists used in the VS validation. (PDF) [file pone.0050816.s001.pdf]

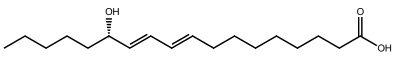

ppar001

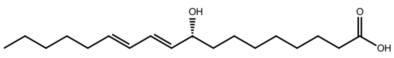

ppar002

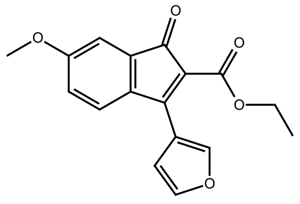

ppar003

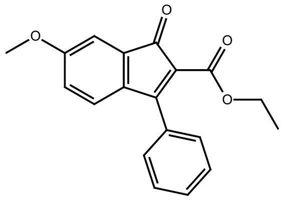

ppar004

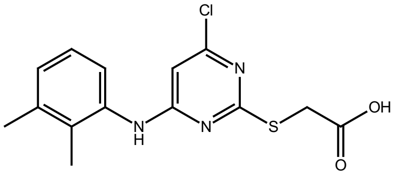

ppar005

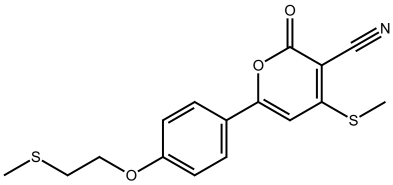

ppar006

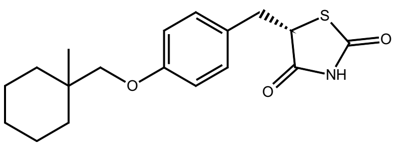

ppar007

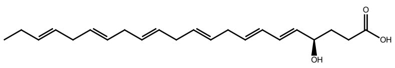

ppar008

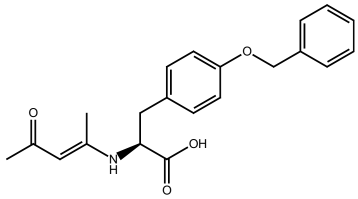

ppar009

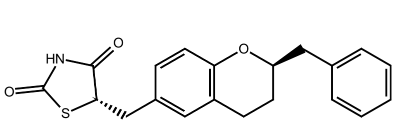

ppar010

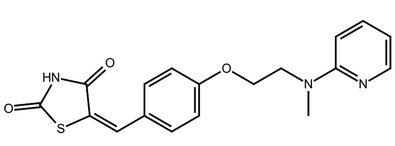

ppar011

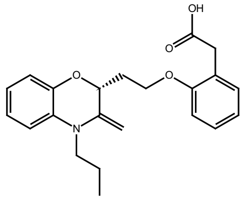

ppar012

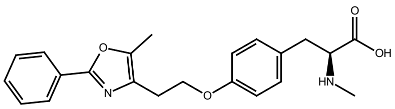

ppar013

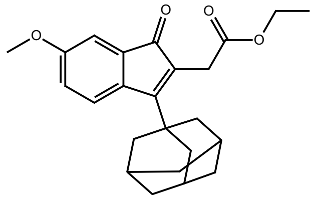

ppar014

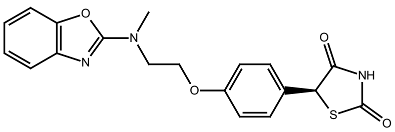

ppar015

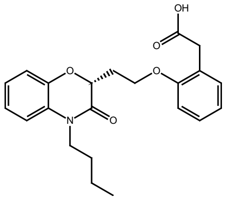

ppar016

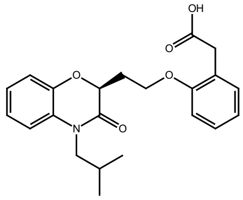

ppar017

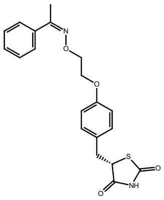

ppar018

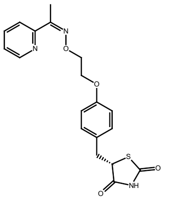

ppar019

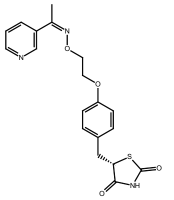

ppar020

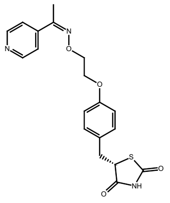

ppar021

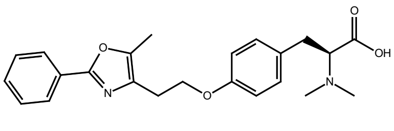

ppar022

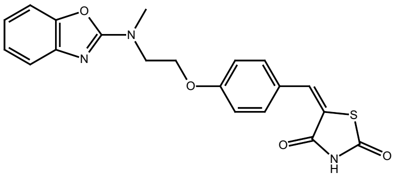

ppar023

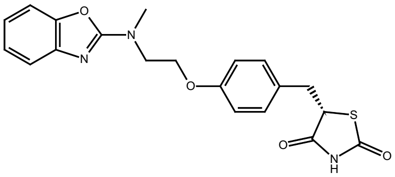

ppar024

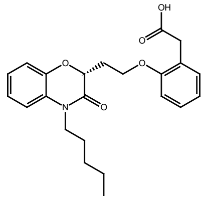

ppar025

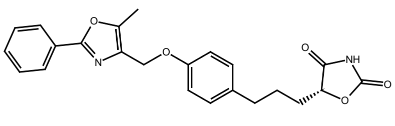

ppar026

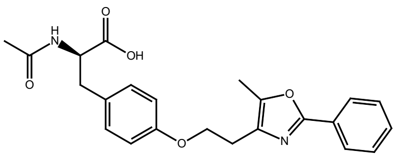

ppar027

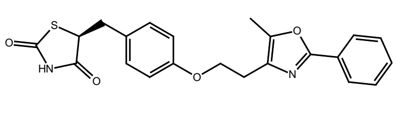

ppar028

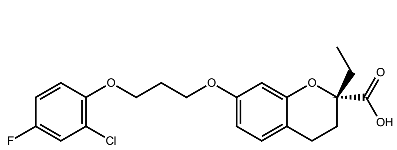
ppar029

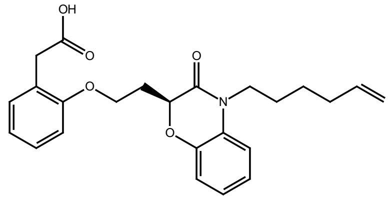
ppar030

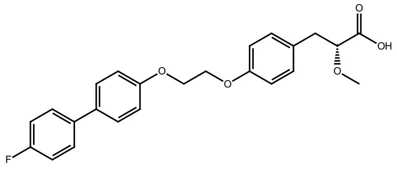
ppar031

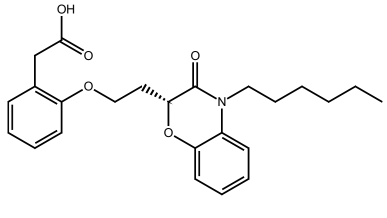
ppar032

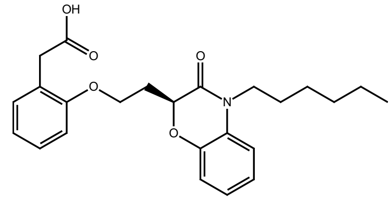
ppar033

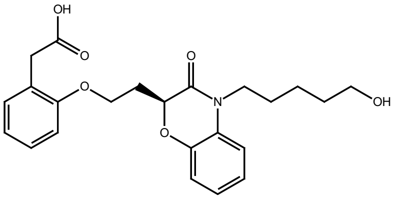
ppar034

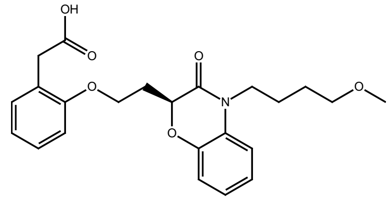
ppar035

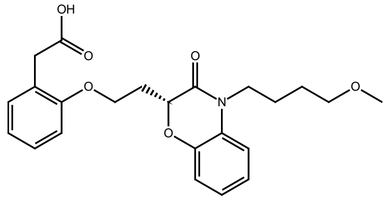
ppar036

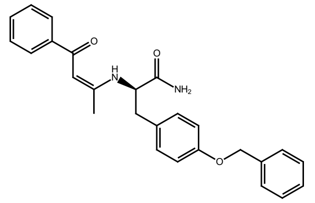
ppar037

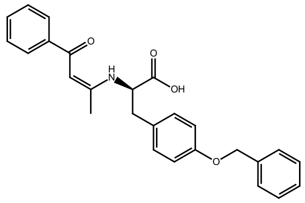
ppar038

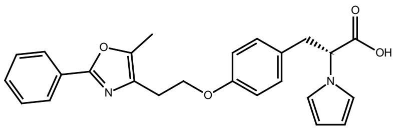
ppar039

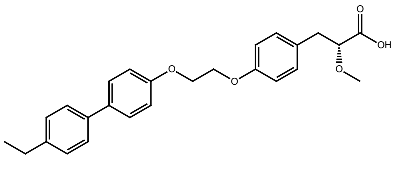
ppar040

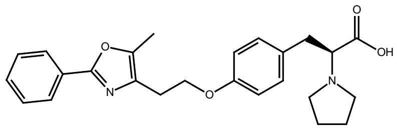
ppar041

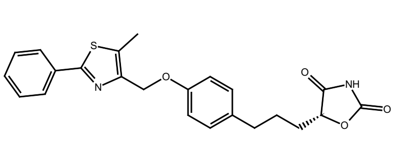
ppar042

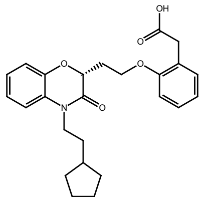
ppar043

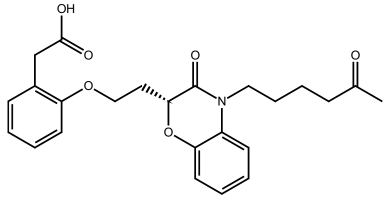
ppar044

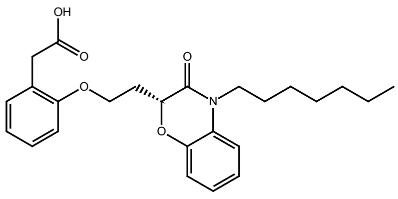
ppar045

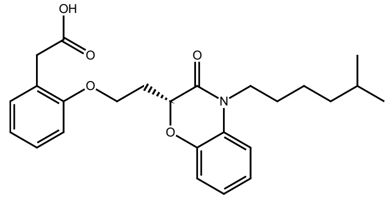
ppar046

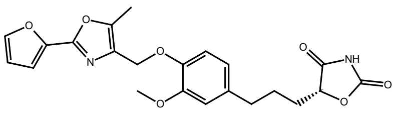
ppar047

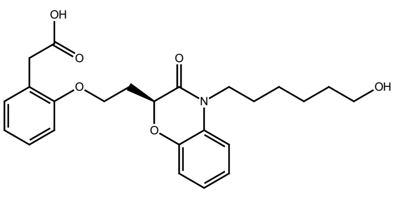
ppar048

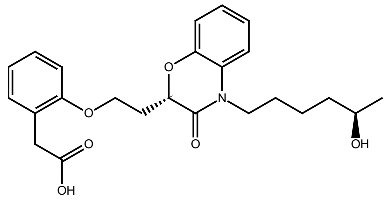
ppar049

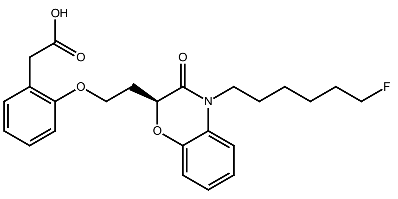
ppar050

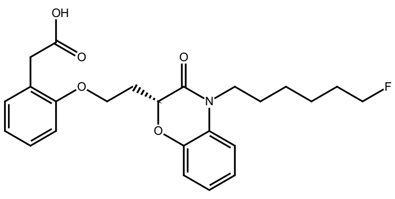
ppar051

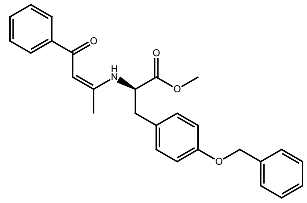
ppar052

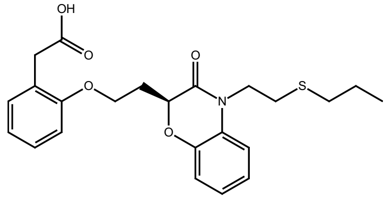
ppar053

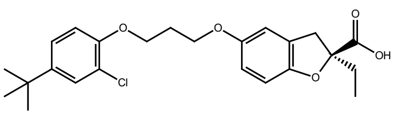
ppar054

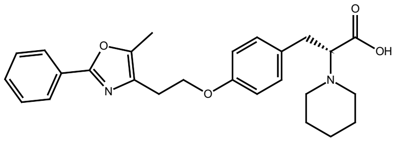
ppar055

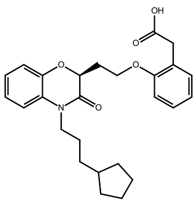
ppar056

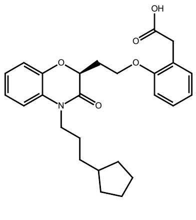

ppar057

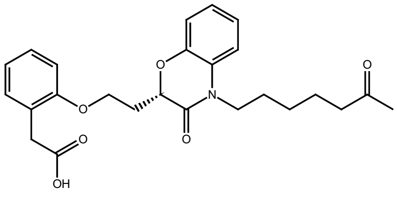

ppar058

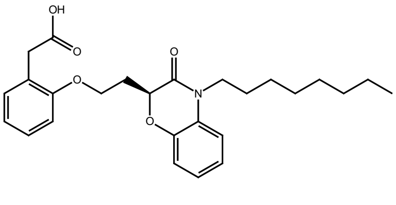

ppar059

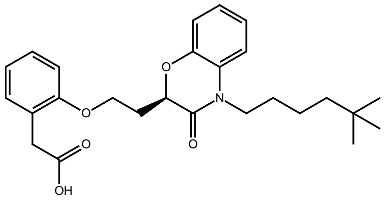

ppar060

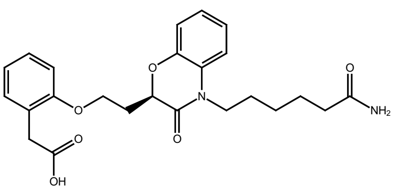

ppar061

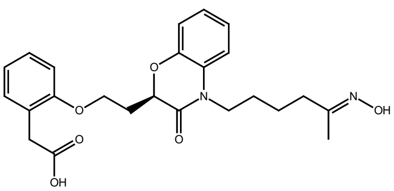

ppar062

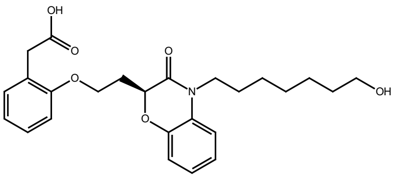

ppar063

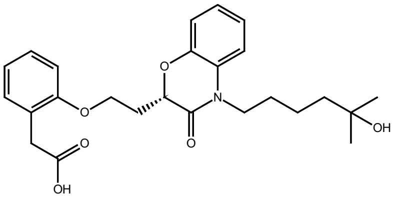

ppar064

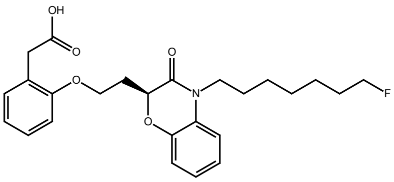

ppar065

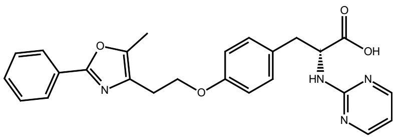

ppar066

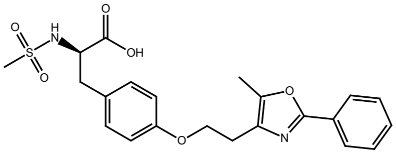

ppar067

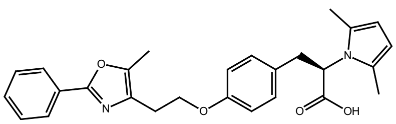

ppar068

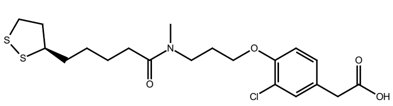

ppar069

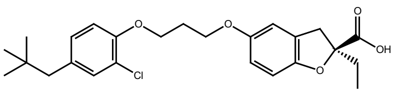

ppar070

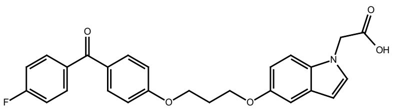

ppar071

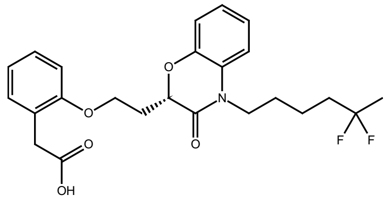

ppar072

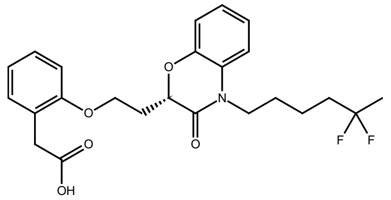

ppar073

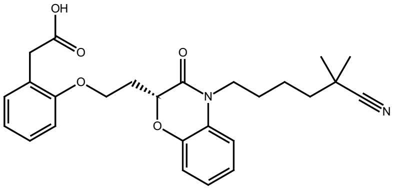

ppar074

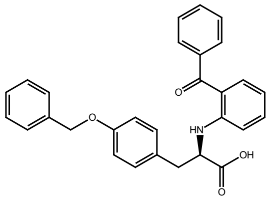

ppar075

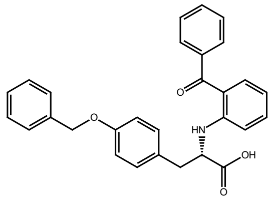

ppar076

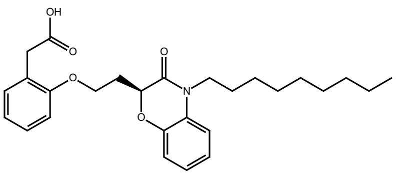

ppar077

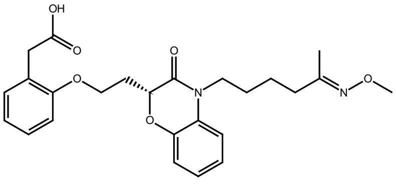

ppar078

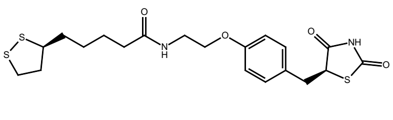

ppar079

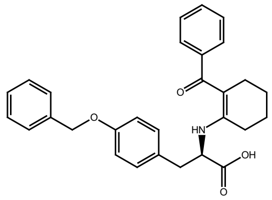

ppar080

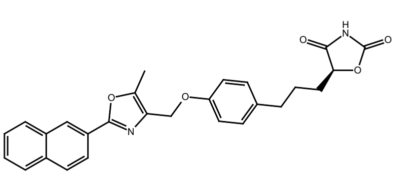

ppar081

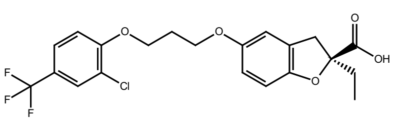

ppar082

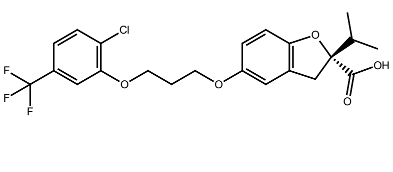

ppar083

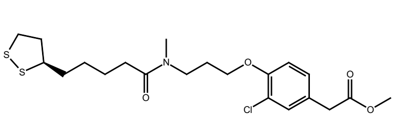

ppar084

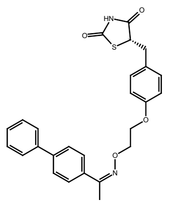

ppar085

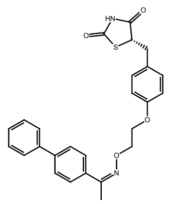

ppar086

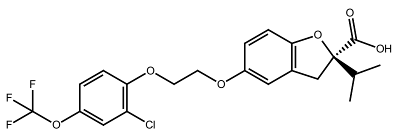

ppar087

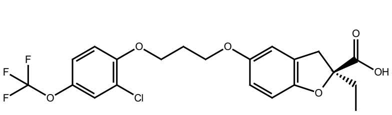

ppar088

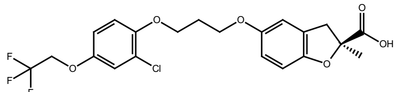

ppar089

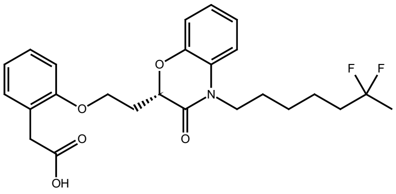

ppar090

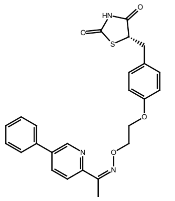

ppar091

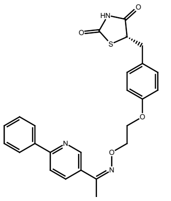

ppar092

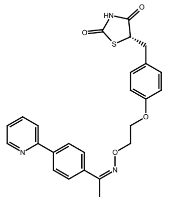

ppar093

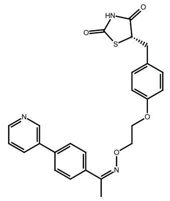

ppar094

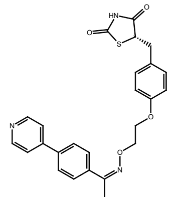

ppar095

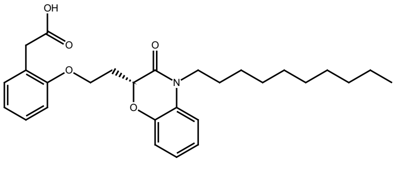

ppar096

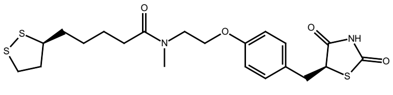

ppar097

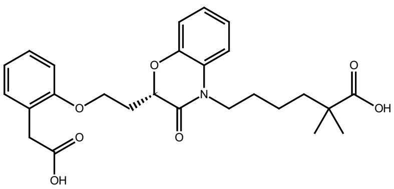

ppar098

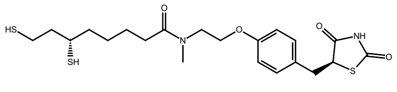

ppar099

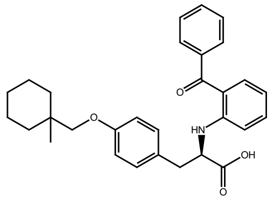

ppar100

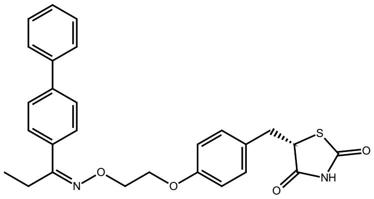

ppar101

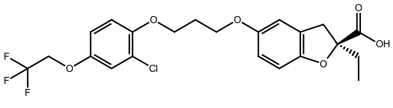

ppar102

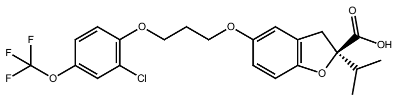

ppar103

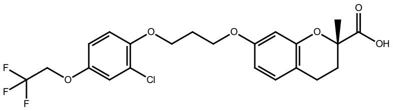

ppar104

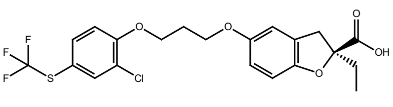

ppar105

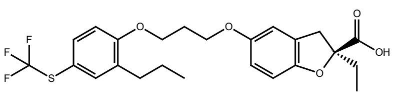

ppar106

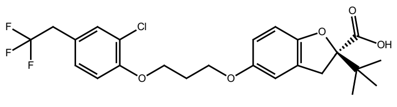

ppar107

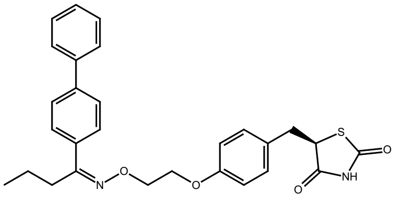

ppar108

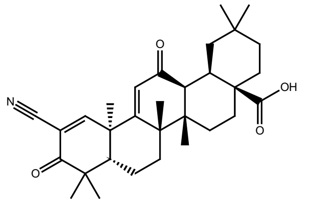

ppar109

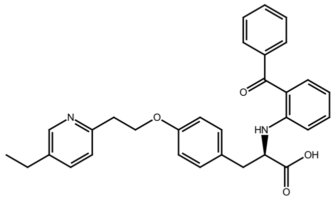

ppar110

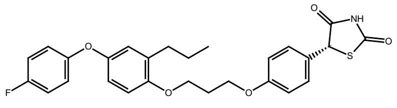

ppar111

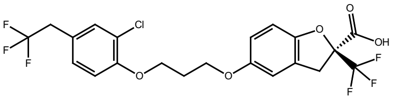

ppar112

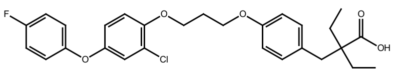

ppar113

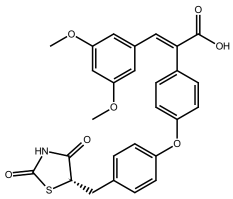

ppar114

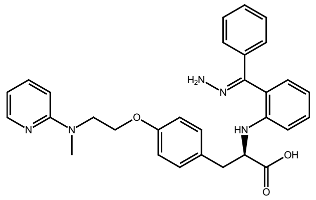

ppar115

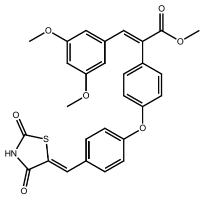

ppar116

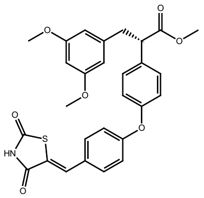

ppar117

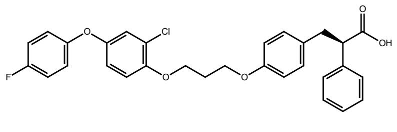

ppar118

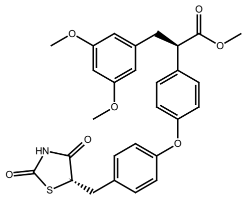

ppar119

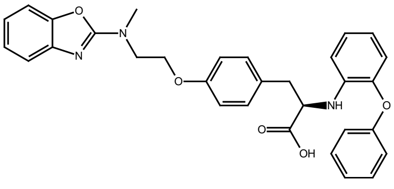

ppar120

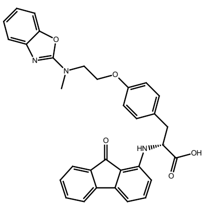

ppar121

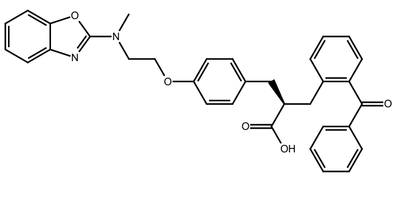

ppar122

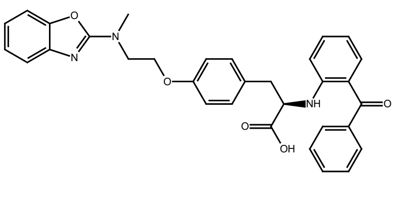

ppar123

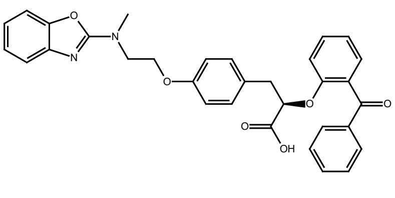

ppar124

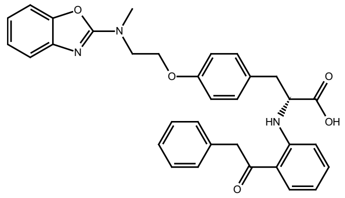

ppar125

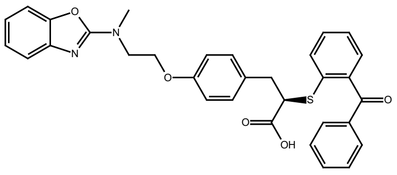

ppar126

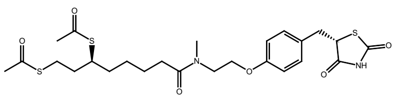

ppar127

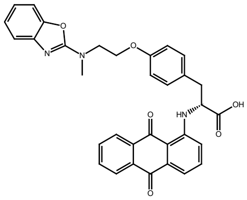

ppar128

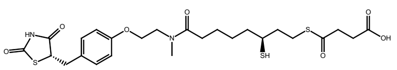

ppar129

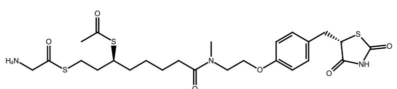

ppar130

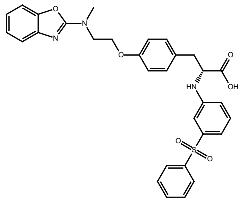

ppar131

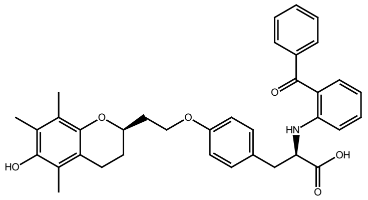

ppar132

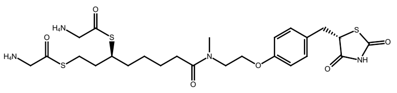

ppar133

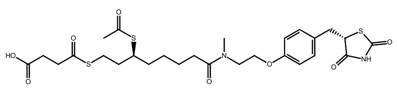

ppar134

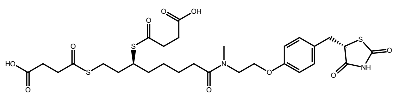

ppar135
