# Supplementary figures and images for: Identification of PPARgamma Partial Agonists of Natural Origin (I): Development of a Virtual Screening Procedure and In Vitro Validation
Source: PLoS One. 2012 Nov 30;7(11):e50816. doi: 10.1371/journal.pone.0050816 (PMC3511273; doi:10.1371/journal.pone.0050816)

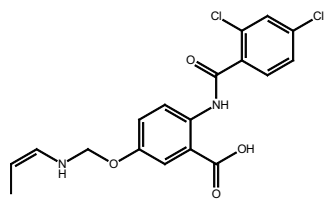

1WMO

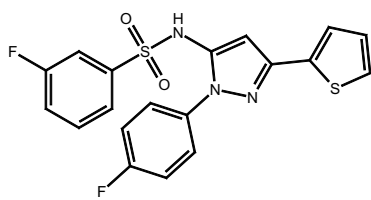

2G0G

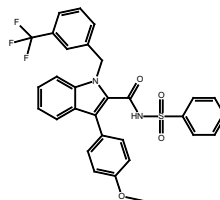

2HFP

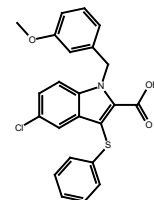

2Q6R

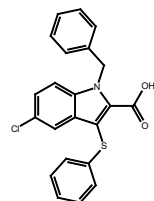

2Q61

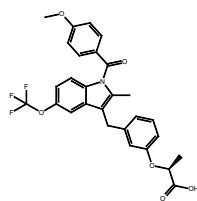

14

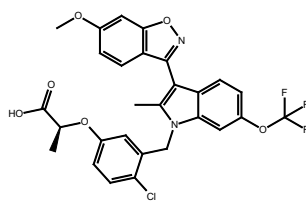

15

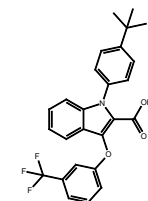

16

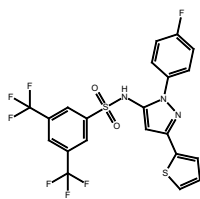

17

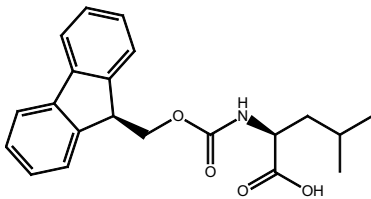

FMOC-L-Leu

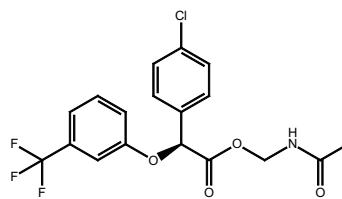

## Metaglidasen

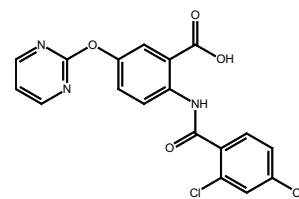

amg-131

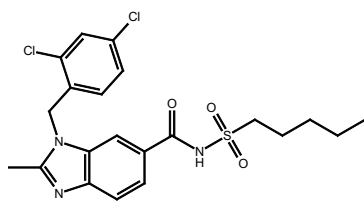

fk-614

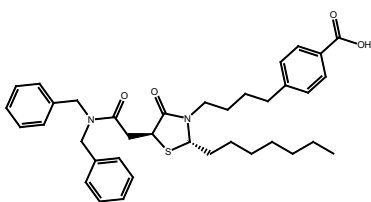

gw0072

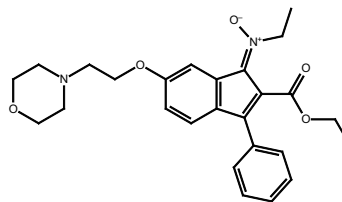

kr-62980

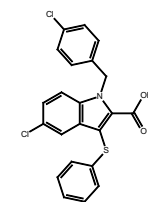

nTZDpa

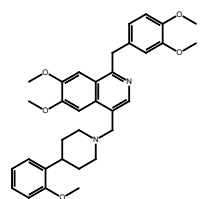

pa-082

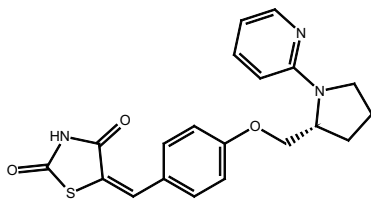

pat5a

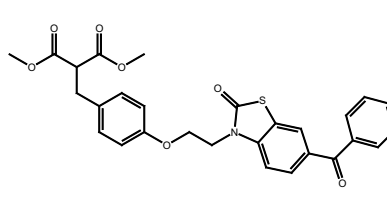

s-26948

Supplement: Table S2 — Structures of the 19 PPARγ partial agonists used in the VS validation. (PDF) [file pone.0050816.s002.pdf]
